# Qingke flavor characteristics and formation mechanisms: volatile metabolites and their changes from raw material to diverse product

**DOI:** 10.3389/fnut.2026.1852893

**Published:** 2026-08-05

**Authors:** Huiyan Xiong, Zhao Liu, Yuanyuan Xie, Mingyang Du, Xuqiu He, Ruijun Duan

**Affiliations:** 1 College of Agriculture and Animal Husbandry, Qinghai University, Xining, Qinghai, China; 2 College of Eco-Environmental Engineering, Qinghai University, Xining, Qinghai, China

**Keywords:** aroma, flavor, formation mechanisms, processing, Qingke, volatile compounds

## Abstract

Qingke (*Hordeum vulgare* L. var. nudum Hook. f.), also known as highland barley, is a distinctive cereal primarily cultivated in the Qinghai-Tibet Plateau region, garnering significant attention for its unique nutritional value and flavor characteristics. With growing demand for healthy foods, Qingke has expanded from a traditional staple into diverse modern product forms like beverages, alcoholic drinks, and condiments. Flavor, specifically aroma, is a critical factor determining consumer acceptability and market competitiveness. Beyond its nutritional value, Qingke possesses a unique and complex flavor profile that underpins its traditional and modern food applications. This review systematically summarizes the flavor features of Qingke and its primary traditional and modern product forms (e.g., Tsamba, Qingke wine, Qingke tea), and delves into the diversity and formation mechanisms of these aroma characteristics. It focuses on analyzing the impact of intrinsic factors (e.g., variety, genetics, environment) and extrinsic factors (e.g., thermal processing, fermentation) on the composition and content of volatile aroma compounds, revealing the pivotal role of aldehydes, alcohols, esters, and heterocyclic compounds (particularly pyrazines) in shaping the unique aroma of Qingke and its products. By comparing the aroma profiles of Qingke, common barley (*Hordeum vulgare* L.) and other cereals, this review identifies Qingke’s composite aroma characteristics, highlighting its unique notes of rice-like, wheaty, sweet, and milky aromas, which are often enhanced by roasted, nutty, fruity, and fermented notes from processing. This comprehensive review aims to establish a solid theoretical foundation for the further development, quality control, and innovative utilization of Qingke and its value-added products, with specific emphasis on future strategies for flavor improvement, standardization, and off-note reduction.

## 1 Introduction

Qingke (*Hordeum vulgare* L. var. *nudum* Hook. f.), commonly referred to as highland barley or Tibetan hullless barley, is a naked barley variety in the Poaceae family. It is named for its naked caryopsis trait, where the grain separates from the lemma, and for its characteristic high-altitude cultivated regions. It is primarily distributed across the Qinghai-Tibet Plateau and surrounding high-altitude regions of China, with a cultivation history dating back approximately 3,500 years (1, 2). As a staple food for local residents, its exceptional abiotic adaptability to harsh high-altitude environments—characterized by low temperatures, intense UV-B radiation, and drought tolerance—has made it indispensable to local agro-pastoral communities (3–5).

In recent decades, Qingke has become a globally recognized “super grain” and a vital “orphan crop” due to its outstanding nutritional properties (6–9). Its nutritional profile significantly surpasses many common cereals and is characterized by high protein, high dietary fiber (especially high *β*-glucan), and high vitamins content, as well as low lipid and low sugar content (10, 11). Qingke boasts a rich protein content (approximately 12–15%), a balanced amino acid composition, and relatively high lysine levels. It serves as an excellent source of dietary fiber and *β*-glucan, the latter of which has been proven to regulate blood sugar and reduce cholesterol effects (12, 13). Additionally, Qingke is abundant in bioactive compounds such as polyphenols (e.g., procyanidin B1, *β*-coumaric acid), *γ*-aminobutyric acid (GABA), and flavonoids, endowing it with remarkable antioxidant, anti-aging, blood sugar and lipid-regulating, glucose metabolism improvement, and immune-enhancing potential (8, 14, 15). These components contribute to its demonstrated health benefits, such as antioxidant, anti-inflammatory, hypoglycemic, and hypotensive activities (16–18). The synergistic effect of procyanidin B1 and *β*-coumaric acid from Qingke in modulating glucose metabolism via the IRS-1/PI3K/Akt pathway underscores its potential in managing metabolic disorders (19).

With the widespread awareness of healthy diet, Qingke has evolved from a regional traditional food into a global recognized healthy food, giving rise to a variety of products. These include traditional staples like Qingke tsamba (roasted barley flour) and Qingke wine, as well as modern products such as Qingke tea (8, 20, 21). Flavor, specifically aroma, is the core determinant of consumer acceptability and market competitiveness. Although the health benefits of Qingke are well-documented, its flavor is equally crucial for consumer acceptance and product diversity. Qingke and its diverse products possess a unique flavor profile distinct from common cereals, which serves as both a source of their appeal and a critical factor in processing and quality control (22–25). Furthermore, the flavor of Qingke is not monolithic; it is a dynamic spectrum that evolves from the raw grain through various processing technologies into a diverse array of products—from the roasted, nutty notes of Qingke tsamba to the complex ester and alcohol profiles of fermented Qingke wine (26–28).

Therefore, systematically analyzing the flavor substance foundation, formation mechanisms, and product manifestations of Qingke is essential for promoting the high-quality development of the Qingke industry. This review aims to provide a comprehensive overview of Qingke flavor research based on existing literature. We will begin by outlining the primary traditional and modern products derived from Qingke and their characteristic flavors. Subsequently, we will analyze the factors shaping its flavor, focusing on two main axes: intrinsic factors (variety differences) and extrinsic factors (processing methods). A major emphasis will be placed on elucidating the formation mechanisms of key flavor compounds, with detailed discussions on the volatile metabolites responsible for its characteristic aroma. Finally, we will summarize the distinctive flavor profile of Qingke, explicitly comparing it with common barley and other cereals to highlight its uniqueness. This review seeks to establish a foundational understanding of Qingke flavor, which is essential for guiding future research, optimizing processing technologies, and the in-depth developing novel, palatable Qingke-based functional foods for a global market.

## 2 Methodology

This study comprehensively reviewed the relevant and contemporary literature on the flavor of Qingke, including Qingke and its products, processing methods, influencing factors, flavor characteristics, and effects on Qingke products. To ensure consistency and repeatability, the precise search methods have been consistently used in different databases, including PubMed, Google Scholar, and Web of Science.

For PubMed, Scopus, and Web of Science, the following search terms and Boolean operators were used: “Qingke flavor “in combination with terms such as “highland barley,” “Tibetan hulless barley,” “Qingke processing products,” “tsamba,” “highland barley wine,” “highland barley tea,” and “flavor,” “flavor compounds “, “volatile metabolites,” “aroma.” The relevance of the title, keywords, and abstract was initially evaluated. Then full-text publications were retrieved and included if they were deemed appropriate for an objective and comprehensive evaluation.

For Google Scholar, the search was performed using the same criteria, but without the ability to apply precise filters such as language or date range. Therefore, the results were manually adjusted based on relevance and date.

The present review utilized peer-reviewed articles and reviews, with a date range spanning from 2000 to 2025, modified according to database specifications.

## 3 Overview of the main Qingke products and their flavor profiles

The processing and utilization of Qingke are diverse, ranging from traditional foods to modern health drinks, from primary products for direct consumption to deep-processed fermented beverages and baked goods, forming a rich product system with distinct flavors. Each product has its own unique flavor profile shaped by the specific biochemical and microbial processes involved.

### 3.1 Traditional staple food: Tsamba (roasted Qingke flour)

Tsamba, namely roasted Qingke flour, is made from Qingke seeds that are washed, roasted, and then ground into flour. In Tibetan culture, Tsamba is considered to be the essence of Tibetan traditional cuisine, the main staple food of Tibetan people, and is often consumed as a power breakfast and light meal. Overall, Tsamba is a versatile and nutritious ingredient that plays a central role in Tibetan cuisine (20, 29).

**Figure 1:**
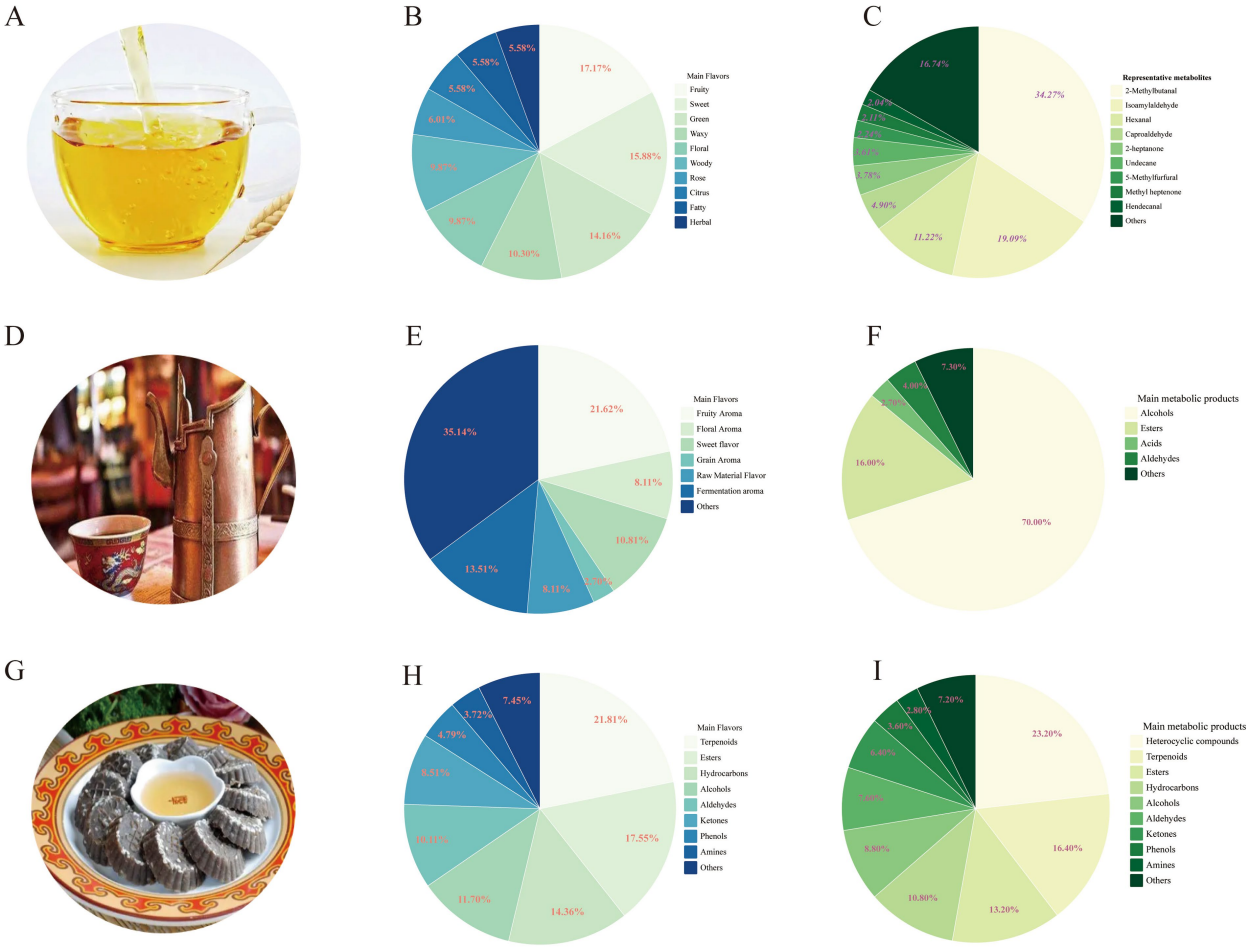
Qingke products and its flavor characteristics. **(A)** Qingke tea products; (B) The main volatile metabolites of Qingke tea; **(C)** Main flavor composition of Qingke tea (our original data); **(D)** Qingke wine products; **(E)** The main volatile metabolites of Qingke wine; **(F)** Main flavor composition of Qingke wine (18, 26, 28, 31). **(G)** Tsamba products; **(H)** The main volatile metabolites of Tsamba; **(I)** Main flavor composition of Tsamba (20, 27).

The roasting process is the critical step in forming Tsamba’s characteristic aroma, which is derived from a series of thermal reactions including the Maillard reaction, Strecker degradation, and lipid oxidation (20, 30). The volatile flavor compounds in Tsamba are dominated by heterocyclic compounds (Figure 1), particularly pyrazines, which constitute 38.59 to 64.42% of the total volatiles across different Qingke varieties. A study using GC-IMS identified 44 volatile organic compounds in Tsamba, including 19 aldehydes, 10 alcohols, 5 ketones, 5 esters, 4 pyrazines, and 1 furan (21, 30). The key aroma-active compounds responsible for the roasted, nutty, and cocoa-like notes are 2,5-dimethylpyrazine, 2,3,5-trimethylpyrazine, 2,3-dimethylpyrazine, and methylpyrazine (20). Additionally, aldehydes such as hexanal and (E)-2-heptenal contribute grassy, green, and fatty notes, while esters like ethyl acetate provide fruity and sweet aromas (25). The sensory profile of roasted Tsamba is characterized by a complex balance of cocoa-like, roasted, nutty, sweet, and green notes (Figure 1), with the roasting temperature significantly influencing the intensity and composition of these flavor attributes (20, 30).

### 3.2 Fermented alcoholic beverage: Qingke wine/Qingke baijiu

Qingke (Highland barley) wine, a traditional fermented beverage from the Qinghai-Tibet Plateau, represents a key deep-processed product of Qingke (Figure 1), including non-distilled fermented wine and distilled (such as Qingke Baijiu) forms. Its complex flavor profile is shaped by a multifaceted microbial metabolic process during fermentation (31, 32). Recent advancements in analytical techniques have significantly progressed research into its flavor composition, formation mechanisms, and influencing factors (28, 33).

The flavor system of Qingke wine comprises volatile and non-volatile compounds. Volatile compounds are central to its aroma, including esters, alcohols, acids, phenols, aldehydes, ketones, and terpenoids (Figure 1) (22, 31). Studies have identified 57 to 66 main flavor substances in Tibetan Qingke wine, with 17 having odor activity values (OAV) above their thresholds, significantly contributing to sensory characteristics like fruity, boiled potato, honey, sweet–sour, and caramel notes (18, 28, 31). Esters (e.g., ethyl acetate, isoamyl acetate) are the most diverse groups, with key components such as ethyl acetate, isoamyl acetate, and ethyl caproate, imparting fruity, floral, and sweet aromas and are crucial for flavor harmony; isoamyl acetate often exhibits the highest OAV (22, 26, 28). Besides ethanol as the base, higher alcohols like isoamyl alcohol and *β*-phenylethanol (OAV up to 452.05) contribute floral and fruity notes (28, 31). Acids, predominantly lactic acid, followed by acetic acid and citric acid, enhance freshness and serve as precursors for ester formation (28, 34). Phenolic compounds (e.g., guaiacol, 4-ethylphenol), derived from the microbial transformation of ferulic acid in Qingke, impart smoky and clove-like aromas (8, 24). Aldehydes such as nonanal and phenylacetaldehyde and terpenoids such as *β*-damascenone, though low in concentration, significantly impact the overall flavor due to their low odor thresholds (25, 28).

### 3.3 Modern drinks (Qingke beverages): Qingke tea

Qingke tea is typically brewed from roasted or fried Qingke seeds (Figure 1). The roasting process (e.g., 170 °C for 10 min) stimulates the Maillard reaction, generating a burnt and nutty aroma while promoting the dissolution of functional components in Qingke (30). Pretreatment methods such as germination, addition of active barley flour, and fermentation can significantly increase the *γ*-aminobutyric acid (GABA) content and antioxidant activity in Qingke beverages, improving their flavor and overall quality (35–37).

For Qingke tea, eight key flavor compounds have been identified: dihydro-2-methyl-3(2H)-furanone (sweet), (E)-2-nonenal (fatty), 2-nonenal (fatty), (E, Z)-2,6-nonadienal (green), 2-phenylethyl 3-methylbutanoate (floral), 4-(2,6,6-trimethyl-1,3-cyclohexadien-1-yl)but-3-en-2-one, dodecanenitrile (citrus), and (E, Z)-2,6-nonadien-1-ol (green) (25, 37) (Figure 1). Existing studies have identified 2-acetyl-1-pyrroline as a key contributor to the popcorn-like scent in roasted Qingke tea, with maltol and isomaltol also recognized as important flavor components (37). Pyrazines (e.g., 2,5-dimethylpyrazine, 2-ethyl-3,5-dimethylpyrazine), furans (e.g., furfural, 5-methylfurfural), pyrroles, and sulfur-containing compounds are major contributors to the roasted aroma (25, 37). Furthermore, numerous previously overlooked trace heterocyclic compounds collectively shape the subtle and complex aromatic profile of Qingke tea (25, 27).

For non-alcoholic beverages, pretreatment methods such as germination and fermentation can significantly increase *γ*-aminobutyric acid (GABA) content and improve flavor and overall quality. The germinated, fermented, and active barley flour-treated (GBTF) samples exhibited the highest GABA content and total phenol content, alongside enhanced antioxidant activity and improved flavor (38).

### 3.4 Other products

#### 3.4.1 Qingke bean paste/vinegar

Qingke bean paste (QBP) is a fermented product produced through the microbial fermentation of Qingke and soybeans. During fermentation, salinity and moisture are key environmental factors that drive the succession of microbial communities, including genera such as Bacillus, Pediococcus, and Issatchenkia. The metabolic activities of these microorganisms generate a variety of volatile flavor compounds, including alcohols, esters, acids, and pyrazines, which collectively form the unique flavor profile of QBP characterized by sauce-like, ester, and umami notes (23). Similarly, the flavor formation of fermented Qingke vinegar is closely associated with the activity of dominant microorganisms (39).

#### 3.4.2 Qingke bread/cake

The incorporation of Qingke flour into baked goods such as bread and cakes often necessitates the use of amendments due to its inherently weak gluten-forming ability. For instance, adding egg powder can improve the quality of Qingke bread by enhancing the structure of the dough (40, 41). Supplementation with an appropriate amount of lysine (e.g., 0.6 g/100 g) can improve the texture of Qingke bread and increase the content of flavor compounds such as ketones and alcohols in the crust, thereby enhancing overall flavor acceptance (42–44). Furthermore, solid-state fermentation of Qingke with different edible mushroom mycelia (e.g., *Morchella esculenta*) can significantly improve its flavor profile when used in baked applications, by reducing grassy odors and generating new aromas such as floral, sweet, and mushroom notes (16).

#### 3.4.3 Qingke beer

The use of roasted Qingke in beer brewing can significantly enhance its flavor profile. Research has shown that beer brewed with Qingke roasted at 180 °C achieves the highest sensory rating. Esters and alcohols constitute the main volatile components in such beer. High-temperature roasting promotes the formation of additional pyrazines and aldehydes, which contribute to the unique flavor characteristics of the beer (17, 45).

## 4 The intrinsic and extrinsic factors influencing Qingke flavor formation

### 4.1 Intrinsic factors

Due to differences in genetic background, planting environment, and metabolite accumulation, different varieties of Qingke exhibit significant variations in the composition of their raw material flavor compounds (46), indicating that the genetic background of Qingke varieties significantly affects their volatile compound composition. An analysis of raw materials from eight different Qingke varieties detected a total of 40 volatile compounds, primarily including aldehydes, alcohols, acids, and ketones. Aldehydes exhibited the highest relative content (ranging from 12.98 to 58.09%) and are an important source of the grassy and fruity flavors characteristic of Qingke raw materials (47).

There are significant differences in the content and proportion of various compounds among different varieties. For example, “Shanqing 9” has the highest aldehyde content (58.09%), while “Zangqing 2000” has the highest acid content (33.90%). Through principal component analysis and cluster analysis, different varieties can be classified based on their flavor composition, providing a basis for screening Qingke raw material varieties for specific flavor needs (47). Another study analyzed 22 varieties of cooked Qingke, identified 44 volatile flavor compounds, and screened for the most suitable variety for cooking applications (21). A detailed flavoromics study of four representative varieties (black, white, blue, yellow) found that black and white Qingke have more intense and complex aroma profiles compared to blue and yellow. This study identified 60 aroma-active compounds and determined that (E)-2-octenal, vanillin, and *γ*-butyrolactone were key contributors to all four varieties, while compounds like 1-pentanol and guaiacol were responsible for aroma differences (22, 25).

Environmental factors, such as high-altitude conditions with intense UV-B radiation, also play a role. The adaptation of Qingke to these environments has led to the accumulation of specific secondary metabolites, which can serve as precursors to unique aroma compounds (3, 22, 25).

### 4.2 Extrinsic factors

Processing is the core link in shaping the final flavor of Qingke products, mainly through two pathways of thermal processing and biological fermentation to trigger complex chemical reactions.

#### 4.2.1 Thermal processing

Thermal processing, including stir-frying, roasting, baking, is the core method for forming the characteristic aroma of products such as tsamba, Qingke tea, Qingke beer, and Qingke bread/cake (17, 27). Research on whole Qingke flour has demonstrated that baking treatment can slow down its flavor deterioration during accelerated storage, whereas puffing treatment can accelerate deterioration (27). Similarly, stir-frying (used for tsamba) and baking (used for beer brewing) generate substantial quantities of pyrazines, aldehydes, and other characteristic flavor substances compounds through thermal processing (17, 20).

**Figure 2:**
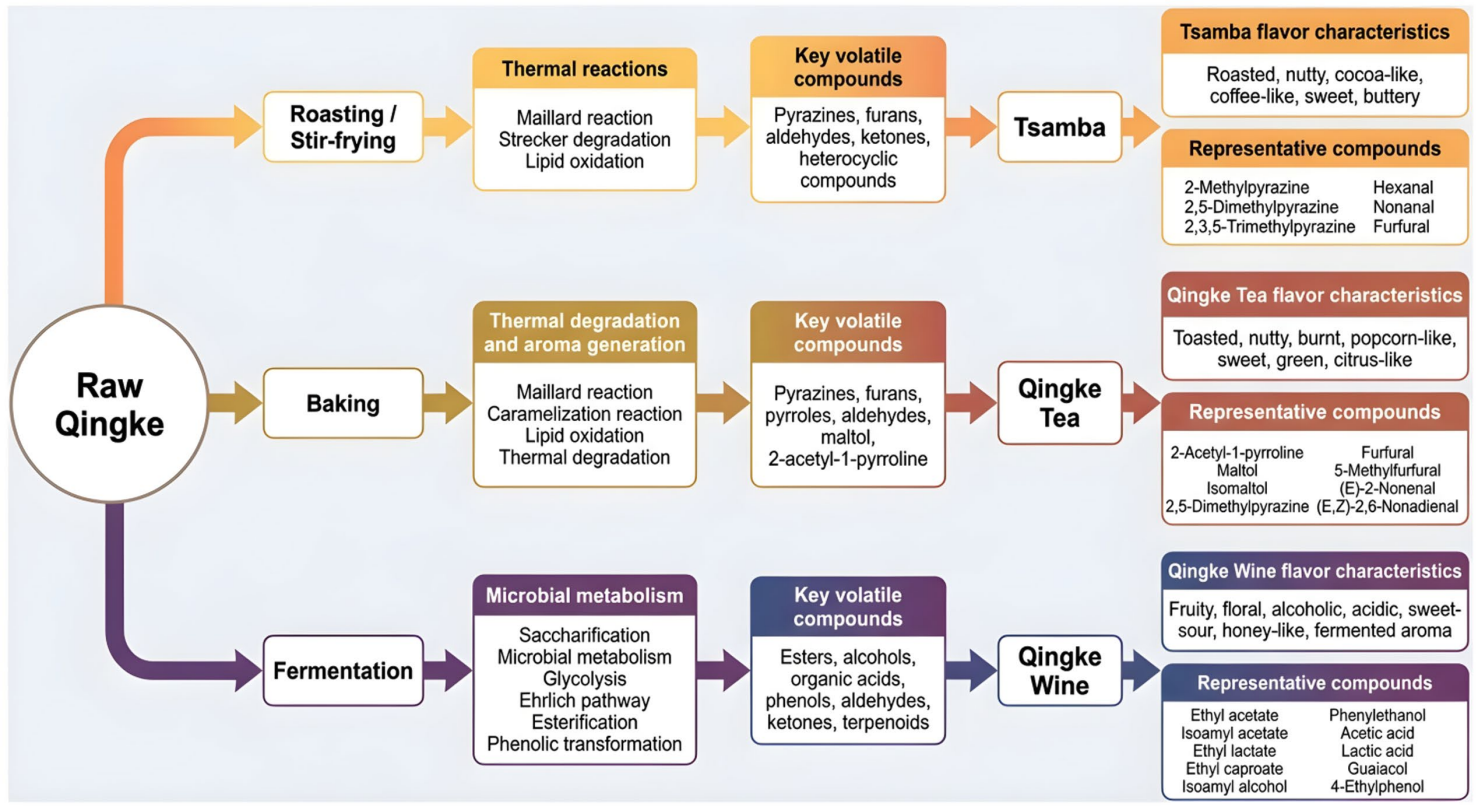
The influence of different processing methods on the key volatile compounds and flavor characteristics of different Qingke products.

At present, research on tsamba mainly focuses on the processing method, with roasting being the key step in forming its characteristic flavor (Figure 2) (29). The roasting process induces a series of thermal reactions, primarily the Maillard reaction and Strecker degradation between reducing sugars and amino acids, alongside lipid oxidation (25, 48). These reactions lead to a dramatic increase in volatile compounds, particularly heterocyclic compounds like pyrazines (e.g., 2-methylpyrazine, 2,5-dimethylpyrazine, 2,3,5-trimethylpyrazine), which are responsible for the characteristic roasty, nutty, cocoa-like, and coffee-like aromas (20, 25). Studies using GC–MS have shown that heterocyclic compounds can constitute 38.59 to 64.42% of the total volatile compounds in roasted Qingke, making them the dominant flavor class (8, 21, 27). Aldehydes, ketones, terpenoids, and esters generated from lipid degradation and other pathways contribute green, fatty, fruity, sweet, and woody notes, adding complexity to the overall profile (30).

Key reactions include the Maillard reaction, Caramelization, and lipid oxidation (Figure 2) (20, 22, 25, 29). In the Maillard reaction, amino acids react with reducing sugars to generate a large number of heterocyclic compounds, especially pyrazines (such as 2-methylpyrazine, 2,5-dimethylpyrazine, 2,3,5-trimethylpyrazine, 3-ethyl-2,5-dimethylpyrazine), which have extremely low thresholds and impart strong baking, cocoa, and nutty aromas (20, 29). Strecker degradation of amino acids further contributes aldehydes and nitrogen-containing heterocycles, enriching the flavor profile (17, 20, 25). In addition, lipid oxidation of unsaturated fatty acids in Qingke produces aldehydes (such as hexanal, nonanal, (E, E)-2,4-decadienal), ketones, and alcohols, contributing grassy, fatty, and fruity notes, with prolonged frying time typically increasing the content of aldehydes and ketones (17, 20). Overall, roasting is an effective method for slowing flavor deterioration, whereas puffing accelerates it (27).

#### 4.2.2 The fermentation process

Fermentation is crucial for products like Qingke wine, Qingke bean paste (QBP), and Qingke vinegar. The fermentation process relies on microorganisms (such as yeast, lactic acid bacteria, and Bacillus) to convert sugars, proteins, and lipids in the raw materials into a rich array of volatile flavor compounds, such as alcohols, esters, acids, and carbonyl compounds (Figure 2) (8, 23, 28, 31, 49). During this process, the sugar metabolism pathway generates ethanol, higher alcohols (e.g., isoamyl alcohol, *β*-phenylethanol), and organic acids (e.g., acetic acid, lactic acid) via glycolysis and the Ehrlich pathway (28, 31, 34). Subsequently, acids and alcohols form esters (e.g., ethyl acetate, ethyl lactate) under enzymatic or chemical action through the esterification pathway, which are the main source of fruity and floral aromas in fermented alcoholic beverages (34). Through amino acid metabolism pathways such as the Ehrlich pathway, amino acids like valine, leucine, and phenylalanine are converted into corresponding higher alcohols and aldehydes (26, 28, 31, 34).

Qingke wine flavor formation is influenced by intrinsic and extrinsic factors. The raw material’s composition is fundamental; Qingke’s high *β*-glucan (3.66–8.62%) and amino acid content provide essential flavor precursors (8, 47). Varietal differences exist, with white six-row barley yielding richer flavors (22). The type of koji (starter) defines the initial microbial community and enzymatic profile, critically influencing flavor diversity. Tibetan koji, containing functional microorganisms like lactic acid bacteria and yeasts, promotes the production of organic acids and esters, contributing to regional character (8, 34, 49). Fermentation process parameters (e.g., koji inoculation rate, temperature, solid–liquid ratio) are also crucial for optimizing flavor compound synthesis (34, 49).

The core formation mechanism involves the transformation of raw material components by microbial metabolism and subsequent chemical reactions. Starch and proteins are degraded into sugars and amino acids by microbial enzymes. Sugars are converted via glycolysis and the acetyl-CoA pathway into ethanol and esters, while amino acids undergo deamination and decarboxylation to form higher alcohols, aldehydes, and ketones (e.g., leucine to isoamyl alcohol) (22, 26, 28, 31, 50). Microbial community succession (dominant genera include Lactobacillus, Pediococcus, Saccharomyces, and Rhizopus) and their metabolic activities are the primary drivers of flavor compound production (23, 34). Non-biological reactions, such as esterification, the Maillard reaction (generating pyrazines for roasted/nutty notes), and oxidation of phenolic compounds, further shape and stabilize the final flavor profile (25, 26, 28, 31).

Fermentation time, temperature, and microbial community structure (e.g., the relative abundance of Lactobacillus, Pichia pastoris, and Rhizopus) directly determine the final flavor profile. Bacillus and Saccharomyces are primarily responsible for volatile aroma compound formation, while Lactobacillus plays a key role in producing acids (23). Salinity and moisture are key environmental factors driving microbial community succession (23). Extending the fermentation period can usually increase the complexity and richness of aromatic substances such as esters (26, 28, 31) (Table 1).

**Table 1:** Aroma characteristics and formation mechanisms across different Qingke products.

Product	Processing type	Aroma characteristics	Representative flavor compounds	Key formation mechanisms	References
Raw material		Grassy, fruity, and variety-dependent flavor differences	Aldehydes, alcohols, acids, ketones; Hexanal, nonanal, benzaldehyde, 1-pentanol	genetic background, growing environment, metabolite accumulation	(20, 22, 25)
Tsamba	Thermal (Roasting)	Roasted, nutty, cocoa-like, sweet	2,5-Dimethylpyrazine, 2,3,5-Trimethylpyrazine, Hexanal, (E, E)-2,4-Decadienal	Maillard reaction, Strecker degradation, lipid oxidation	(20, 27, 30)
Qingke Wine	Biological (Fermentation)	Fruity, floral, alcoholic, sweet–sour, acidic, complex, and rich aroma	Isoamyl alcohol, *β*-Phenylethanol, Ethyl acetate, Ethyl lactate, 4-Vinylguaiacol	Microbial metabolism (glycolysis, Ehrlich pathway), esterification	(8, 18, 26, 31, 34, 45)
Qingke Tea	Thermal (Roasting)	Roasted, nutty, popcorn-like, burnt	Dihydro-2-methyl-3(2H)-furanone, 2-Acetyl-1-pyrroline, 2,5-Dimethylpyrazine	Maillard reaction, caramelization	(22, 25, 30, 37)
Qingke Bean Paste	Biological (Fermentation)	Sauce-like, ester, umami, nutty	2,5-Dimethylpyrazine, 2,3,5-Trimethylpyrazine, 2,3-Butanediol	Microbial metabolism (Maillard reaction, esterification)	(23)

## 5 The flavor characteristics of Qingke and its comparison with barley and other cereals

### 5.1 Flavor composition and perception in Qingke

The aroma of Qingke and its products is the result of the combined action of numerous volatile compounds. Through techniques such as gas chromatography–mass spectrometry (GC–MS), gas chromatography-olfactometry (GC-O), aroma extract dilution analysis (AEDA), and odor activity value (OAV) calculations, key compounds that contribute significantly to the overall aroma of Qingke have been identified (22, 25).

Aldehydes are usually present in high content and contribute greatly to the volatile profile of Qingke. They are abundant in raw grains and many processed products, and are important contributors to grassy, fatty, and fruity flavors. Compounds such as hexanal (grassy), (E, E)-2,4-decadienal (oily), and benzaldehyde (nutty) often have high OAVs and are important sources of the characteristic grain and green grass aroma in Qingke (8, 20, 22, 24). Alcohols are particularly prominent in fermented products like wine. Compounds like isoamyl alcohol (alcoholic, fruity), *β*-phenylethanol (rosy), and 1-hexanol (grassy) have a significant impact on the aroma of wine (8, 25, 34). Esters are considered the “soul” of fermented products, contributing fruity and floral aromas. Their key components include ethyl acetate (fruity), ethyl lactate (milky, fruity), and ethyl caproate (pineapple-like) (8, 16, 17, 34). Heterocyclic Compounds (especially Pyrazines) are characteristic flavor substances of thermally processed Qingke products (e.g., tsamba), providing strong roasted, nutty, and cocoa aromas. Their content and variety are important indicators for evaluating the degree of roasting or baking (20, 24, 25, 27). Ketones and Lactones compounds such as 2,3-butanedione (buttery), *γ*-nonalactone (milky, coconut), and γ-valerolactone (milky) contribute to the sweet and creamy sensory characteristics of some Qingke products (22). Phenols and aromatic substances like 4-vinylguaiacol (smoky, clove-like) and 2-acetylthiazole (rice-like) impart unique baking and cereal aromas to Qingke products, commonly found in fermented and certain thermally processed products (22, 25).

The perception of flavor is not simple the superposition of individual volatile compounds, but rather the specific perception of many volatile compounds through complex interactions. Research has shown there are synergistic, additive, or masking effects among volatile compounds. For example, in Qingke aroma recombination experiments, a synergistic effect was observed between vanillin (sweet) and *γ*-nonalactone (milky), while 2-pyrrolidone, 2-acetylthiazole, 4-vinylguaiacol, and hexanal exhibited masking effects on the aroma of the former pair. These interactions profoundly affect the overall flavor profile of Qingke (22). Furthermore, the absolute content of a volatile compound is not always proportional to its aroma contribution. The odor activity value (OAV, i.e., concentration divided by odor threshold) is the key indicator for measuring its sensory impact. Some volatile substances with high content but a high threshold (e.g., certain long-chain fatty acids) may contribute very little, while some trace volatile compounds with extremely low thresholds (e.g., *β*-diketones) can have a substantial impact on the overall flavor (20, 22, 25).

### 5.2 Comparison of flavors between Qingke and common barley (*Hordeum vulgare* L.)

Comparative research provides an important perspective for understanding the uniqueness of Qingke aroma (25). As a naked grain variety of barley, Qingke shares both similarities and differences in flavor with common barley (hulled barley, *Hordeum vulgare* L.). Both share many basic cereal aroma components. Sensory analysis indicates that the flavor profiles of Qingke and barley are primarily characterized by rice-like, wheat-like, sweet, and milky notes. Key aroma-active compounds such as 2,3-butanedione (creamy), 2-pyrrolidone (wheat-like), *γ*-nonalactone and γ-valerolactone (milky), vanillin (sweet), and hexanal (grassy) have been identified in both and contribute significantly to the overall aroma (high FD values). These shared components form a similar aromatic background, reflecting their close genetic and metabolic backgrounds (22, 24, 25).

Regarding the flavor differences between Qingke and barley, Vanillin (sweet, vanilla) is considered a key differential marker, with its content in ordinary barley (877.01 μg/kg) typically higher than in Qingke (466.1 μg/kg) (22, 25). And flavoromics study detected 50 aroma-active compounds in Qingke compared to 38 in barley. Qingke also possesses unique key aroma components, such as (E, E)-2,4-decadienal, methional, phenylacetaldehyde, and 4-hydroxybenzaldehyde, which may be related to the accumulation of secondary metabolites from its adaptation to high-altitude environments (22). Descriptive analysis suggests that Qingke’s rice-like and sweet aromas are often perceived as more prominent. In fermented beverages, Qingke can impart a distinctive “cereal freshness” and “clean sweetness” compared to traditional barley beer or whiskey (28).

The flavor of black and white Qingke is often perceived as richer and more complex than some barley varieties. In products like liquor (QKH) brewed from high-altitude Qingke, most aroma compounds exhibit higher OAVs. Qingke’s unique varieties (e.g., black, purple) and traditional processing methods (e.g., tsamba roasting, specific wine fermentation) jointly shape its distinctive flavor profile (8, 20, 22, 34).

### 5.3 Comparison with other cereals

To strengthen this work, this review also compared Qingke with other major cereals. The key aroma compound in fragrant rice, 2-acetyl-1-pyrroline, is also an important component in Qingke tea. However, Qingke has a broader profile including wheaty and milky notes which are less common in rice (51, 52). Wheat aroma is characterized by a strong cereal, bread-like, and sometimes nutty profile from compounds like 2-pyrrolidinone, which is also found in Qingke. Unlike wheat, Qingke possesses a more distinct milky and sweet profile, potentially due to higher levels of lactones (25, 51, 52).

Oats have a distinct creamy, fatty, and sometimes grassy aroma profile. The industrial processing of oats, specifically kiln-drying, generates pyrazines and furans via Maillard reactions, imparting nutty and roasted notes, a process very similar to Qingke roasting (53). However, Qingke’s aroma profile appears more diverse due to its wider range of traditional and modern product forms and the unique metabolites from its high-altitude adaptation (Table 2).

**Table 2:** Effects of thermal processing vs. fermentation on key aroma compound classes in Qingke.

Compound class	Raw material	Thermal processing	Fermentation
Aldehydes	High, main source of grassy note	Increase significantly; main products of lipid oxidation & Strecker degradation (e.g., hexanal, nonanal, (E, E)-2,4-decadienal)	Moderate formation; mainly from amino acid metabolism
Esters	Low	Limited change; not dominant	Increase significantly; become major contributors to fruity/floral notes (e.g., ethyl acetate, isoamyl acetate)
Alcohols	Low to moderate	Slight increase; minor contribution	Increase significantly; include ethanol and higher alcohols (e.g., isoamyl alcohol, β-phenylethanol)
Pyrazines (Heterocycles)	Very low	Increase significantly; become key compounds (e.g., 2,5-dimethylpyrazine), contributing roasted, nutty aromas	Moderate increase; can be formed via Maillard reaction or microbial activity
Organic Acids	Low	Moderate increase	Increase significantly; contribute to sourness and act as ester precursors (e.g., lactic acid, hexanoic acid)

## 6 Conclusion and prospect

This review summarizes the complex flavor system of Qingke, which is shaped by a matrix of intrinsic (variety, genetics, environment) and extrinsic (thermal processing, fermentation) factors (20, 25). The fundamental flavor profile of Qingke is characterized by cereal, wheat-like, sweet, and milky notes, which are shared with but distinct from ordinary barley in terms of key aroma composition and sensory intensity. Aldehydes, ketones, lactones, and phenolic compounds form the key volatile backbone of Qingke’s flavor (22). Its unique flavor identity is defined by specific aldehydes and differential markers like vanillin content (22, 25). For example, there are clear differences exist between Qingke and common barley, particularly in the concentration of key differential markers like vanillin and in the overall diversity and intensity of aroma-active compounds.

Processing methods significantly expand this flavor spectrum, generating rich roasted, nutty, and cocoa aromas dominated by pyrazine compounds from thermal reactions, complex fruity, floral, and alcoholic notes by ester, and phenolic from microbial fermentation (1, 8, 16, 20, 34). The formation of these flavors results from the interplay of genetic traits, raw material composition, processing techniques, and biochemical reactions (Maillard reaction, lipid oxidation, microbial metabolism) (20, 34). Volatile metabolites such as aldehydes, alcohols, esters, and heterocycles are the final chemical expressions, creating unique sensory profiles through specific concentrations and interactions (22).

Despite significant progress in understanding the aroma chemistry of Qingke, several critical bottlenecks remain. To advance from descriptive analysis to predictive and controllable flavor design, we propose the following specific, actionable research routes.

First, future research should focus on flavor standardization and off-note reduction through precise processing optimization. And processing parameters (e.g., frying temperature/time, fermentation strains) need optimization based on flavor formation mechanisms to achieve standardized and controllable flavor profiles (27). For thermal processing (e.g., roasting tsamba), the Maillard reaction time–temperature curve needs optimization to maximize desirable pyrazines (roasted, nutty) while minimizing bitter aldehydes from excessive lipid oxidation. A key strategy is developing a “flavor index” based on OAV ratios—for example, the ratio of vanillin (sweet) to hexanal (grassy) could serve as a quantitative quality control metric for tsamba (20). To reduce grassy off-notes in fermented products, controlled inactivation of the lipoxygenase (LOX) pathway prior to processing (e.g., via brief blanching) could be explored, allowing thermal reactions to dominate the final aroma profile (27). Additionally, screening for starter cultures that suppress grassy notes and the use of enzyme treatments such as lipoxygenase inhibitors should be further investigated to control lipid-derived aldehyde formation.

Second, deciphering variety-specific and microbially-driven flavor formation requires deeper mechanistic study. Deeper mechanistic study is required to understand how genetic background and microbial ecology dictate flavor outcomes. The metabolic profiles of aroma compounds across different Qingke varieties (e.g., black, white, blue) are not fully clarified, hindering flavor-oriented breeding (21). Furthermore, the dynamic correlation between microbial communities and flavor metabolite formation during fermentation requires investigation using integrated multi-omics approaches—combining metagenomics, metatranscriptomics, and metabolomics. This systems-level approach can identify key enzymes and pathways responsible for key esters and higher alcohols, enabling more precise microbial community engineering for flavor control (23, 26, 32, 54).

Third, a multi-technique analytical framework is essential to overcome the limitations of individual methods. HS-SPME-GC–MS (Headspace Solid-Phase Microextraction Gas Chromatography–Mass Spectrometry) is excellent for identifying a wide range of volatiles but is semi-quantitative, whereas GC-O with AEDA (Gas Chromatography-Olfactometry with Aroma Extract Dilution Analysis) directly links compounds to sensory perception but is time-consuming. GC-IMS (Gas Chromatography-Ion Mobility Spectrometry) offers rapid, high-sensitivity screening but provides less definitive compound identification (30). A recommended workflow is: (1) rapid screening using GC-IMS or e-nose; (2) comprehensive identification using GC–MS with GC-O and AEDA; (3) rigorous quantification and OAV (Odor Activity Value) calculation; and (4) sensory validation through aroma recombination and omission experiments (20, 22, 25, 55).

Fourth, the synergy between health benefits and flavor quality represents a major untapped opportunity. Qingke’s renowned health benefits (high *β*-glucan, low GI) have been emphasized, but how these nutritional components influence flavor formation remains underexplored. The following issues should be considered: (a) how β-glucan’s viscosity alters volatile release kinetics in the mouth; (b) how the amino acid profile determines Maillard reaction outcomes (generating pyrazines); (c) how phenolic compounds act as precursors for volatile phenols (e.g., 4-vinylguaiacol) while also contributing bitterness and astringency; and (d) how the starch structure influences texture and perceived sweetness (28). Simultaneous optimization of health functionality and flavor quality is needed to enhance consumer acceptance of functional Qingke foods (11).

In summary, future research should integrate metabolomics, microbiology, and sensory science to construct a full-chain flavor regulation strategy from “raw material → processing → microbe → metabolite → sensory perception” (22). A systematic understanding of Qingke flavor will not only aid in preserving traditional food cultures but also provide a scientific basis for developing novel health foods and beverages, enabling this “super grain” to thrive in broader markets (28).
